# Reliability of Magseed® marking before neoadjuvant systemic therapy with subsequent contrast-enhanced mammography in patients with non-palpable breast cancer lesions after treatment: the MAGMA study

**DOI:** 10.1007/s10549-024-07407-6

**Published:** 2024-06-20

**Authors:** Eva Iglesias Bravo, Antonio Mariscal Martínez, Helena Peris Alvà, Diego Riol Sancho, José Carlos Antela López, Joel Aranda Sánchez, Pilar Escobar Casa, Cristina Gómez de las Heras, María Antonia Fernández Venegas, Eduarda García Vidal, Elisabeth Delgado Begines, Carmen García Mur, Isabel Vicente, Carmen Casamayor, Silvia Cruz, Anabel García Barrado

**Affiliations:** 1 Obstetrics and Gynaecology Department, Virgen de Valme University Hospital, Seville, Spain; 2 Breast Diagnostic Imaging Unit (BDIU) Department of Radiology, Hospital Universitari Germans Trias i Pujol (HUGTiP), Badalona, Barcelona, Spain; 3 Canary Islands University Hospital Complex - Materno Infantil de Canarias (CHUIMI), Canaria University Hospital, Las Palmas, Spain; 4 Radiology Department, Virgen de Valme University Hospital, Seville, Spain; 5 Radiology Department, Miguel Servet University Hospital, Saragossa, Spain; 6 Gynaecology Department, Miguel Servet University Hospital, Saragossa, Spain; 7 Surgery Department, Miguel Servet University Hospital, Saragossa, Spain; 8 Servicio de Obstetricia y Ginecología, Hospital Universitario Virgen de Valme, Ctra. de Cádiz Km. 548,9, 41014 Seville, Spain

**Keywords:** Breast cancer, Magnetic seed, Neoadjuvant systemic therapy, Non-palpable lesions, Surgery

## Abstract

**Purpose:**

To assess the reliability of excising residual breast cancer lesions after neoadjuvant systemic therapy (NAST) using a previously localized paramagnetic seed (Magseed®) and the subsequent use of contrast-enhanced spectral mammography (CESM) to evaluate response.

**Methods:**

Observational, prospective, multicenter study including adult women (> 18 years) with invasive breast carcinoma undergoing NAST between January 2022 and February 2023 with non-palpable tumor lesions at surgery. Radiologists marked tumors with Magseed® during biopsy before NAST, and surgeons excised tumors guided by the Sentimag® magnetometer. CESMs were performed before and after NAST to evaluate tumor response (Response Evaluation Criteria for Solid Tumors [RECIST]). We considered intraoperative, surgical, and CESM-related variables and histological response.

**Results:**

We analyzed 109 patients (median [IQR] age of 55.0 [46.0, 65.0] years). Magseed® was retrieved from breast tumors in all surgeries (100%; 95% CI 95.47–100.0%) with no displacement and was identified by radiology in 106 patients (97.24%), a median (IQR) of 176.5 (150.0, 216.3) days after marking. Most surgeries (94.49%) were conservative; they lasted a median (IQR) of 22.5 (14.75, 40.0) min (95% CI 23.59–30.11 min). Most dissected tumor margins (93.57%) were negative, and few patients (5.51%) needed reintervention. Magseed® was identified using CESM in all patients (100%); RECIST responses correlated with histopathological evaluations of dissected tumors using the Miller–Payne response grade (*p* < 0.0001) and residual lesion diameter (*p* < 0.0001). Also 69 patients (63.3%) answered a patient’s satisfaction survey and 98.8% of them felt very satisfied with the entire procedure.

**Conclusion:**

Long-term marking of breast cancer lesions with Magseed® is a reliable and feasible method in patients undergoing NAST and may be used with subsequent CESM.

**Supplementary Information:**

The online version contains supplementary material available at 10.1007/s10549-024-07407-6.

## Background

Breast cancer (BC) is increasingly diagnosed at early stages, when lesions are still small and non-palpable, owing to the implementation of screening programs and improved detection methods [1, 2]. Moreover, neoadjuvant systemic therapy (NAST) in advanced BC allows to decrease tumor size before surgery and increase the options for breast-conserving surgery [3, 4]. Consequently, surgeons increasingly face the challenge of accurately excising small, non-palpable tumors while removing the smallest amount of healthy tissue for optimal clinical and cosmetic outcomes [5].

Accurate excision of non-palpable tumors in conservative surgery requires its precise intraoperative location. Wire-guided localization (WGL) is a safe, cost-effective, well-established technique and the gold standard [6]. However, it has disadvantages, such as patient discomfort and pain, and has been associated with hematoma development and vasovagal reaction in some patients [6–8]. Moreover, the wire is usually placed on the same day of surgery, contributing to patients’ distress and limiting efficiency in the operating room and scheduling flexibility [6, 8, 9]. Wire placement may be challenging and compromise incision positioning and surgical dissection, resulting in suboptimal cosmetic outcomes and non-target tissue removal [7, 8, 10, 11].

Alternative tumor localization methods have been developed to overcome the limitations of WGL, such as radioactive seed localization, radioguided occult lesion localization, and intraoperative ultrasound, but their disadvantages have precluded their widespread use [6]. Magseed® is a paramagnetic seed for breast tumor and axillary lymph node localization that may be placed any time before surgery, with proven safety and effectiveness before, after, and during NAST [6]. Magseed® is not displaced and it is identified using mammography and ultrasound, independently of the nuclear medicine department. The surgery team can locate the seed intraoperatively using the Sentimag® probe, which transiently magnetizes it [12]. This method overcomes limitations regarding operation room logistics (by decoupling radiology and surgery), incision placement, and marker identification [12]. Overall, studies have reported successful tumor localization, Magseed® identification and retrieval, and optimal surgical outcomes [12]. For lymph node localization in targeted axillary dissection (TAD) before and after NAST, Magseed® has shown good results owing to its small size [13–15].

Magseed® may be placed during biopsy before NAST, minimizing patient discomfort. However, paramagnetic seeds interfere with magnetic resonance imaging (MRI), the gold standard imaging method to assess patients’ response to NAST in the breast, challenging the evaluation of residual breast disease [16]. Contrast-enhanced spectral mammographies (CESMs) have emerged as an alternative imaging method for tumor evaluation [17] and have shown high concordance rates with MRIs at diagnosis and follow-up of patients receiving NAST [18–20]. Moreover, CESMs have demonstrated the ability to detect residual disease after NAST and predict complete pathological response with high sensitivity and negative predictive value. Furthermore, evaluation of NAST responses using CESM and pathological criteria (i.e., Miller and Payne) are highly concordant [18–24].

Despite the increasing number of observational studies assessing Magseed®, the reliability of Magseed® marking before NAST to guide excision of resulting non-palpable tumors with subsequent CESM to evaluate the presence of residual disease after NAST has not been reported [25]. This observational, prospective, study aimed to assess the reliability of excising non-palpable breast tumor lesions after NAST using a previously localized magnetic seed (Magseed®) and the subsequent use of CESM to evaluate response. Secondary objectives were to assess Magseed® in TAD and patients’ satisfaction with the procedure.

## Methods

### Study design and population

This observational, prospective, multicenter study (MAGMA) included adult women (> 18 years) with invasive breast carcinoma with an indication for NAST. Patients were consecutively recruited between January 2022 and February 2023 from the Gynecology and Radiology departments in four participating centers in Spain: Hospital Universitario Virgen de Valme (Sevilla), Hospital Universitario Miguel Servet (Zaragoza), Hospital Universitari Germans Trias i Pujol (Badalona), and Hospital Universitario Materno-Infantil de Gran Canaria (Las Palmas de Gran Canaria). Results from the Hospital Universitari Germans Trias i Pujol (Badalona) patient series will be published separately elsewhere [26]. Lesions were required to be non-palpable at the time of surgery after NAST. Patients who did not provide written informed consent and those with previous disease, metallic prosthesis, extensive microcalcifications, inflammatory carcinoma, and clinical situations suggestive of radical surgery, such as high tumor-to-breast ratio, suspected poor clinical response, and metastatic carcinoma, were excluded from the study. Radiologists marked patients’ tumors with Magseed® (Endomag, UK) guided by ultrasound during tumor tissue biopsy before NAST. Breast tumor tissue was retrieved during surgery guided by the Sentimag® magnetometer system (Endomag, UK). Contrast-enhanced mammographies were performed before and after NAST to evaluate tumor response. Magseed ® was also placed in 36 patients’ lymph nodes distributed in all participating centers. Figure S1 includes a diagram of the study design.

This study was conducted according to the principles of the Helsinki Declaration (Fortaleza, Brazil) and local data protection regulations (2016/679 and 14/2007). All patients provided written informed consent to participate in the study. The local Ethics Commitees of the four participating centers approved this study’s protocol.

### Objectives, assessments, and variables

For the primary objective (i.e., reliability of dissecting residual breast lesions after NAST marked with Magseed® before NAST and subsequent use of CESM to evaluate response), we assessed Magseed®-related intraoperative variables, including Magseed® retrieval (yes/no), displacement (yes/no), identification by radiological examination (X-ray) within the dissected breast tumor specimen (yes/no), and correlation with CESM (yes/no). We also considered the type of surgery (conservative/mastectomy), surgery time (min), and surgical outcomes (positive margins, reintervention, and residual tumor diameter [mm]).

The primary objective also included the reliability of subsequent CESMs to evaluate tumor response to the therapy. CESM-related variables were Magseed® identification using CESM before and after NAST (yes/no) and responses to NAST based on Response Evaluation Criteria for Solid Tumors v1.1 (RECIST v1.1) (disease progression, stable disease, partial response, and complete response) [27]. The reliability of the RECIST response based on CESM imaging was evaluated by assessing correlations with histological response, including Miller–Payne grades (G1 to G5), residual tumor diameter (mm), positive margins (yes/no), and reintervention (yes/no). Additional histopathology variables assessed were histological type (ductal, tubular, and others), tumoral grade (I, II, and III), tumoral phenotype (HER2, luminal A, luminal B, luminal B HER2, and triple-negative), and Tumor Node Metastasis (TNM) classification system. Other CESM-related variables were identification projection and background enhancement pattern before and after NAST, as well as reduction (no reduction, size reduction, and both size and background enhancement pattern reduction) and type of reduction (concentric, disappearance, and patchy) after NAST.

Secondary variables were Magseed® retrieval from axillary lymph nodes and correspondence between marked and sentinel lymph nodes. Additional secondary objectives were professionals’ and patients’ satisfaction with the technique. Gynecologists and surgeons reported their satisfaction with cosmetic results (yes/no) and the easiness of Magseed®-guided surgery (yes/no). Patients answered a 4-item satisfaction survey designed ad hoc for this study, including the following questions: (1) The use of the tumor localization technique with the Magseed® magnetic seed placed before starting neoadjuvant chemotherapy treatment avoids the need to place a WGL on the same day of surgery. How effective do you find this localization technique? (Please choose a score between 1 and 7); (2) If you have experienced discomfort/pain during or after the Magseed®/harpoon placement, please rate the intensity on a 1–7 scale (1, no pain; 7, severe pain); (3) If the procedure has caused any adverse effects, please rate the intensity level (1, mild intensity; 7, high intensity); and (4) Overall, your satisfaction with the entire procedure is: (please choose a score between 1 and 7).

Baseline patients’ demographics (age) and clinical characteristics (body mass index, menopause, and tumor characteristics) were collected from medical records. Baseline tumor characteristics included TNM grade, main lesion size (mm), main lesion BI-RADS (Breast Imaging-Reporting and Data System) category, and staging. Variables related to tumor and axillary lymph nodes marking before NAST were Magseed® localization in breast tumors, positive lymph nodes (yes/no) and number, marked positive lymph nodes (yes/no), and lymph node marker used.

### Statistical analysis

With a sample of 100 patients, the unilateral 97.5% confidence interval (CI) range based on the Clopper-Pearson method would be 7.3% for sensitivity and 19.9% for specificity, with a lower CI limit for sensitivity of 87.7% and a lower CI limit for specificity of 75.1%.

Categorical variables were described as frequencies and percentages, and continuous variables as the median and interquartile range (IQR: Q1, 25th percentile, and Q3, 75th percentile). No imputation method was used for missing data. Correlations between RECIST and histological responses (Miller–Payne grade and diameter of the residual lesion) were assessed by calculating Spearman’s rank correlation coefficient. Statistical significance was set at a two-sided α < 0.05. All statistical analyses were performed using SAS® version 9.4.

## Results

### Characteristics of study patients and Magseed® marking

Of the 131 patients recruited, 109 were evaluable and included in the analysis (Figure S2). Median (IQR) age was 55.0 (46.0, 65.0) years, and most women (60.55%) were menopausal. Patients’ demographic, clinical, and tumor characteristics are summarized in Table 1. Patients’ tumors were most frequently T2 (78.90%), N1 (48.15%), and M0 (99.07%), and of BI-RADS category 5 (58.72%).

**Table 1:** Demographic, clinical, and breast cancer characteristics of study patients, n = 109

Demographic	
Age (years), median (IQR)	55.0 (46.0, 65.0)
Clinical	
Body mass index (kg/m^2^), median (IQR)	27.7 (24.4, 32.0)
Menopause, n (%)	
Yes	66 (60.55)
No	43 (39.45)
Breast cancer	
TNM grade, n (%)	
cT	
T0	0 (0)
T1	9 (8.26)
T2	86 (78.90)
T3	13 (11.93)
T4	1 (0.92)
cN, n = 109	
N0	50 (45.87)
N1	52 (47.71)
N2	7 (6.42)
N3	0 (0.00)
Main lesion size (mm), median (IQR) n = 109	
Anteroposterior axis	25.00 (18.75, 32.00)
Craniocaudal axis	24.00 (18.00, 32.00)
Left–right axis	24.00 (17.00, 32.25)
Main lesion BI-RADS category, n (%)	
0	1 (0.92)
1	0 (0.00)
2	0 (0.00)
3	0 (0.00)
4-A	2 (1.83)
4-B	8 (7.34)
4-C	29 (26.61)
5	64 (58.79)
6	5 (4.59)
Staging, n (%) n = 109	
0	0 (0)
IA	0 (0)
IB	8 (7.34)
IIA	53 (48.62)
IIB	33 (30.28)
IIIA	9 (8.26)
IIIB	3 (2.75)
IIIC	2 (183)
IV	1 (0.92)

Unless otherwise stated

*BI-RADS* breast imaging-reporting and data system, *IQR* interquartile range, *TNM* tumor node metastasis

Magseed® was localized in a central location in the tumor tissue in most cases (n = 80, 76.92) and was retrieved a median (IQR) of 176.5 (150.0, 216.3) days after marking (Table S1). Over half (n = 59, 54.13%) of the patients had affected lymph nodes; most patients with affected lymph nodes had only one affected lymph node; and the majority (77.97%) of them were marked, mostly with Magseed® (91.31%).

### Magseed® retrieval, displacement, and surgery outcomes

Magseed® was retrieved from breast tumor tissue in all surgeries (n = 109 [100%]; 95% CI 95.47–100.0%) and was not found displaced from the breast lesion in any case (Table 2). It was identified in visible breast dissected tissue in most cases (n = 106, 97.24%) by radiological examination. The three (2.75%) patients with unavailable data were due to seed loss in the operating room before radiological examination.

**Table 2:** Magseed® retrieval and displacement from breast tissues, n = 109

Breast surgical specimen	
Seed retrieval, n = 109	
n (%)	109 (100.0)
95% CI (%)	95.47‒100.0
Seed displacement	
Correlation with CESM, n (%) n = 24	24 (100)
Displacement within the breast lesion, n (%) n = 109	
Yes	0 (0)
No	109 (100)
Confirmation of the seed in surgical visible specimen, n = 106	
Yes	106 (97.24)
No	3 (2.75)

*LN* lymph node, *CESM* contrast-enhanced spectral mammography, *CI* confidence interval

Magseed® was identified in all axillary lymph nodes (n = 42, 100%) and was retrieved from all the dissected ones (n = 42, 100%) (Table S2).

Most surgeries (n = 103, 94.49%) were conservative, and only six (5.51%) were mastectomies (Table 3). Surgeries (n = 80 with available data) were performed in a median (IQR) of 22.5 (14.75, 40.0) min (95% CI 23.59–30.11). Most dissected tumor margins (93.57%) were negative, with a median (IQR) residual lesions diameter of 10.0 (0.0, 18.5) mm (95% CI 9.75–15.51), and few (n = 6, 5.51%) patients needed reintervention (Table 3). Histopathological characteristics of dissected tumors are summarized in Table S3.

**Table 3:** Surgery characteristics and outcomes, n (%) n = 109

Type of surgery, n = 109	
Conservative	103 (94.49)
Mastectomy	6 (5.51)
Positive margins, n = 109	
Yes	7 (6.43)
No	102 (93.57)
Need for reintervention, n = 109	
Yes	6 (5.51)
No	103 (94.49)
Residual lesion diameter (mm), n = 92	
Median (IQR)	10.0 (0.0, 18.5)
95% CI	(9.75–15.51)

*CI* confidence interval, *IQR* interquartile range

### Outcomes related to contrast-enhanced mammography

Magseed® was identified using CESM in all patients (100%) irrespective of the different enhancement patterns before and after NAST and the different tumor reduction types (Table 4).

**Table 4:** Magseed® identification using contrast-enhanced spectral mammography before and after neoadjuvant systemic therapy (NAST), n (%)

Before NAST, n = 109	
Successful Magseed® identification	109 (100)
Identification projection	
Craniocaudal	0 (0.00)
Mediolateral oblique	1 (0.93)
Both	108 (99.07)
Background enhancement pattern, n = 101	
No enhancement	12 (11.88)
Minimum	59 (58.42)
Moderate	30 (29.70)

After NAST, n = 109	
Successful Magseed® identification	109 (100)
Identification projection	
Craniocaudal	0 (0.00)
Mediolateral oblique	1 (0.92)
Both	108 (99.08)
Background enhancement pattern	
No enhancement	33 (30.28)
Minimum	62 (56.88)
Moderate	14 (12.84)
Reduction, n = 108	
Both (size and background enhancement pattern)	94 (87.04)
No reduction	3 (2.78)
Size	11 (10.19)
Type of reduction, n = 105	
Concentric	49 (46.67)
Disappearance	48 (45.71)
Patchy	8 (7.62)

Missing data for one patient

*NAST* neoadjuvant systemic therapy

Of the 109 patients, 51 showed a complete response, 45 showed a partial response, 10 showed stable disease, and three showed disease progression. Responses according to RECIST criteria correlated with responses evaluated by histopathology in the dissected tumors using the Miller–Payne response grade (*p* < 0.0001) and residual lesion diameter (p < 0.0001) (Table 5). Patients with complete response showed higher response grades (G > 3) more frequently (93.88%) than those with stable disease and disease progression (0% for both), who showed G ≤ 3 responses more frequently (100% for both). Most patients with a partial response showed G3 responses (64.4%). Similarly, the residual lesion diameter progressively decreased with improved radiological response, from a median (IQR) of 38.0 (37.5, 46.5) mm in patients with disease progression to 0.0 (0.0, 3.5) mm in those with complete response. Regarding positive margins and reintervention, few (n = 7) patients had positive margins, six of whom needed reintervention, all with stable disease or partial response, whereas no patients with complete response had positive margins or reintervention (Table 5).

**Table 5:** Correlation between radiological response (RECIST criteria) and histological response and positive margins, n = 106

	Disease progression	Stable disease	Partial response	Complete response	*p*-value^a^
Miller–Payne response grade, n	3	9	45	49	< .0001
G1	3 (100.00)	1 (11.11)	0 (0.00)	0 (0.00)	
G2	0 (0.00)	3 (33.33)	5 (11.11)	0 (0.00)	
G3	0 (0.00)	5 (55.56)	29 (64.44)	3 (6.12)	
G4	0 (0.00)	0 (0.00)	8 (17.78)	15 (30.61)	
G5	0 (0.00)	0 (0.00)	3 (6.67)	31 (63.27)	
Residual lesion diameter (mm), n	3	9	40	40	
Mean (SD)	43.33 (10.12)	30.33 (16.12)	16.01 (10.95)	2.98 (5.70)	< .0001
Median (IQR)	38.0 (37.7. 46.5)	25.0 (20.0. 36.0)	14.0 (10.0. 21.0)	0.0 (0.0. 3.5)	
95% CI	(31.89–54.78)	(19.80–40.87)	(12.61–19.40)	(1.21–4.74)	
Positive margins, n	2	9	43	49	N/A
Yes	0 (0.00)	0 (0.00)	7 (16.28)	0 (0.00)	
No	2 (100.00)	9 (100.00)	36 (83.72)	49 (100.00)	
Need for reintervention, n	2	9	45	48	N/A
Yes	0 (0.00)	1 (11.11)	5 (11.11)	0 (0.00)	
No	2 (100.00)	8 (88.89)	40 (88.89)	48 (100.00)	
Positive margins or reintervention, n	2	9	43	48	N/A
Yes	0 (0.00)	1 (11.11)	7 (16.28)	0 (0.00)	
No	2 (100.00)	8 (88.89)	36 (83.72)	48 (100.00)	

*IQR* interquartile range, *N/A* not applicable, *RECIST* response evaluation criteria in solid tumors, *SD* standard deviation

^a^Spearman rank test

^b^Missing

Data regarding CESM tumor evaluation before and after NAST are summarized in Tables 6 and 7.

**Table 6:** CEDM results prior to surgery, n = 107

Tumour size anteroposterior axis (mm)	
Mean (SD) 95% CI (%) Median (Min; Max) P25; P75	30.3 (13.1) 27.8‒32.8 27.0 (0.0; 70.0) (21.0; 37.0)

Tumour size craniocaudal axis (mm)	
Mean (SD)	28.3 (12.6)
95% CI (%)	25.8‒30.7
Median (Min; Max)	26.0 (0.0; 80.0)
P25; P75	(20.0; 34.0)

Tumour size left–right axis (mm)	
Mean (SD)	27.5 (10.8)
95% CI (%)	25.4‒29.5
Median (Min; Max)	25.0 (0.0; 55.0)
P25; P75	(18.0; 36.0)

*SD* standard deviation, *CI* confidence interval, *P* percentile, *RECIST* response evaluation criteria in solid tumors

Missing data in two patients

**Table 7:** CEDM results post-NAST reduction, n = 108

Both	92 (86.8)
Size	11 (10.4)
No reduction	3 (2.8)
Enhancement	0 (0.0)
Missing	2
Type of reduction, n = 108	
Concentric	48 (46.6)
Disappearance	47 (45.6)
Patchy	8 (7.8)
Missing	5
Tumour size (mm), n = 108	
Anteroposterior axis	
Mean (SD)	10.1 (12.8)
95% CI (%)	7.6‒12.5
Median (Min; Max)	6.0 (0.0; 64.0)
P25; P75	(0.0; 16.0)
Craniocaudal axis	
Mean (SD)	9.7 (12.8)
95% CI (%)	7.2‒12.1
Median (Min; Max)	6.5 (0.0; 71.0)
P25; P75	(0.0; 15.0)
Left–right axis	
Mean (SD)	9.0 (11.6)
95% CI (%)	6.8‒11.3
Median (Min; Max)	6.0 (0.0; 63.0)
P25; P75	(0.0; 15.0)
RECIST response grade, n = 108	
Complete response (CR) (100%)	50 (46.7)
Partial response (PR) (30–99%)	44 (41.1)
Stable disease (SD) (< 30% or increases < 20%)	10 (9.3)
Disease progression (DP) (increases > 20%)	3 (2.8)
Missing	1

*SD* standard deviation, *CI* confidence interval, *P* percentile, *RECIST* response evaluation criteria in solid tumors

Missing data in one patient

### Healthcare professionals’ and patients’ satisfaction

Gynecologists and surgeons were satisfied with the cosmetic results (n = 77 of 78, 98.72%) and with the easiness of Magseed®-guided surgery (n = 77 of 79, 97.47%).

A total of 65 (60.2%) patients answered the patients’ satisfaction questionnaire. Most gave the maximum score (7 points, 84.6%) to tumor marking before NAST with Magseed® compared to the use of WGL during surgery (Table S4). Moreover, patients mostly reported low pain levels after Magseed® (≤ 2, 81.5%) and due to adverse events (≤ 2, 92.3%). Almost all patients scored the entire procedure ≥ 6 points (of a maximum of 7) (n = 64, 98.4%).

## Discussion

This observational, prospective study (MAGMA study) provides evidence regarding the reliability of long-term marking with Magseed® to guide excision of non-palpable BC lesions after NAST and the subsequent use of CESM to evaluate tumor response. Magseed® was successfully retrieved from breast tumors in all surgeries and identified in almost all cases by radiological examination, with no displacement in any case. In all cases, surgeons identified Magseed®; in a few cases, no identification was due to specimen deterioration during surgery. Likewise, surgeons retrieved it from all the marked and dissected axillary lymph nodes. Most surgeries were conservative, mostly with negative tumor margins, and few patients needed reintervention. CESM allowed Magseed® identification before and after NAST in all patients, and responses according to RECIST criteria, assessed using CESM, correlated with histological responses evaluated in dissected tumors with Sensibility of 80.70%, Specificity 93.87%, Negative Predictive value of 80.70%, and Positive Predictive Value of 93.87% (Table 8). Gynecologists and surgeons were satisfied with the cosmetic results and the ease of Magseed®-guided surgery overall, and almost all patients scored their satisfaction with the entire procedure with the highest scores.

**Table 8:** Matrix comparing response by RECIST and Miller–Payne n = 106

		Response by Pathology Miller–Payne		Sum
		Response	No response	
RECIST complete response	Yes	46	3	49
	No	11	46	57
		57	94	106
Sensitivity		46/(46 + 11)	80.70%	
Specificity		46/(46 + 3)	93.87%	
Positive predictive value		46/(3 + 46)	93.87%	
Negative predictive value		46/(46 + 11)	80.70%	

Data were missing for 3 patients

Magseed® is a radiation- and wire-free localization method that has been shown to successfully locate BC lesions in several previous retrospective and prospective studies [12, 15, 25, 28–30]. However, the populations included in these studies were heterogeneous regarding the characteristics of tumors (palpable vs. non-palpable) and setting (NAST vs. no NAST), and, more importantly, pre-operatively localized Magseed® was assessed, within a few days before BC surgery, unlike this study assessing long-term marking before NAST, hindering direct comparisons [12]. Despite this fundamental difference, results from this study were similar regarding successful Magseed® localization, tumor identification and excision, with low positive margins (6.8% in this study and ranging 0‒16.0% in previous studies) and reintervention rates (5.7% in this study vs. 11.19% in previous studies), supporting its use for long-term marking in the NAST setting [12, 15, 25, 28, 30–34]. Moreover, other studies and pooled analyses comparing Magseed® with WGL showed similar clinical outcomes regarding positive margins (16.0% for Magseed® vs. 14.0% for WGL) and reintervention rates (11.25% for Magseed® vs. 16.4‒20.0% for WGL) [12, 32].

Tumor localization based on radioactivity, including radioactive seed localization and radioguided occult lesion localization, has shown comparable results to WGL. They are well-established methods for long-term marking and have become standard in some countries [5, 6]. However, in other countries, the use of radioactivity has not been approved or it is subject to complex radiation safety regulations, precluding their routine use and increasing costs [6, 35]. Moreover, radioactivity-based methods involve professionals from nuclear medicine departments and radiologists, surgeons, and gynecologists. In this context, this study provides evidence of long-term tumor marking using an alternative wire-free method that overcomes the limitations associated with nuclear isotopes [6].

In this study, the seed remained in place and was successfully retrieved from all patients, despite seed location in tumors for a median of 176.5 days (approximately six months) before surgery, during biopsy before starting NAST. A previous retrospective study in a smaller cohort showed similar results for Magseed® for an average of 138 days after localization, with all seeds being retrieved and confirmed in the specimen [25]. In this previous study, 10.0% of patients had positive margins, a higher proportion than in our study (6.8%). Moreover, we showed that marking metastatic axillary lymph nodes with Magseed® at the time of biopsy allowed their successful identification and excision, as shown in a previous study [13]. These results indicate that Magseed® is a useful method for long-term marking of BC lesions and axillary lymph nodes and, importantly, enables biopsy and marking in one procedure, minimizing patients’ discomfort. In this regard, NAST is increasingly used in the management of BC patients, and therefore, more patients may benefit from the advantages of Magseed® localization at the time of biopsy [36].

Despite the advantages of Magseed® localization during biopsy before starting NAST, magnetic seeds interfere with MRIs, precluding evaluation of the tumor response [16]. For this reason, we evaluated RECIST responses based on CESM and showed a significant correlation with histopathological responses in tumor specimens dissected with Magseed® guidance, supporting the reliability of CESM to assess response shown in previous studies [18–23]. Moreover, Magseed® was identified using CESM in all patients before and after NAST. These results indicate that CESM may be the alternative to MRI for tumor imaging and response evaluation in patients with a localized paramagnetic seed before NAST. The simultaneous use of Magseed® and CESM enabled the marking of BC lesions during biopsy before starting NAST and evaluation of tumor responses during patients’ follow-up. Even though MRIs are the gold standard for patients’ follow-up, the availability of MRI equipment may be low in low-income settings and varies among and within countries. It is an expensive and time-consuming imaging technique and it is not applicable to patients with contraindications [37–39]. Moreover, multiple specialties share MRI equipment available at hospitals, and its use is not exclusive to breast units. In contrast, CESM is being increasingly incorporated as a diagnostic technique in Breast Radiology units, and gynecologists and oncologists are its main users, making CESM equipment more accessible than MRI equipment [40, 41]. CESM facilitates scheduling and reduces the workload of radiology services and waiting times, providing advantages for MRI technicians, breast cancer surgeons, and patients.

Healthcare professionals and patients were highly or very highly satisfied with the procedure using Magseed®. Except for one isolated case during the initial learning curve, they were all satisfied with the cosmetic results and the ease of Magseed®-guided surgery, in line with previous studies reporting that healthcare professionals rated favorably the method’s feasibility and easiness over WGL [42–44]. Moreover, patients were highly or very highly satisfied with the procedure, particularly regarding overall satisfaction and the effectiveness of the localization procedure not being performed on the same day of surgery.

The results from this study should be interpreted in the context of general and specific limitations. Although the study’s sample size was sufficient to assess Magseed® reliability, the resulting low positive margins and low reintervention rates precluded establishing clear correlations with RECIST responses. Despite its limited sample size, the study included patients treated in four different centers, capturing the experiences with Magseed® in four public university hospitals across Spain. In this regard, large academic hospitals have a high patient volume and number of surgeries and, therefore, may have increased expertise with Magseed® than smaller centers. Consequently, results from this study may not be generalizable to smaller centers performing fewer surgeries and with a slower learning curve. Nevertheless, minimal technical challenges and a learning curve with Magseed® have been previously reported [30]. Furthermore, given its prospective nature, missing data were minimal, except for 39.8% of patients who did not answer the questionnaire. Despite these limitations, this study showed that the use of Magseed® with subsequent CESM is a feasible and reliable option for long-term tumor marking and follow-up of patients receiving NAST.

## Conclusions

Long-term marking of breast cancer lesions at the time of biopsy with the paramagnetic seed Magseed® is a reliable, feasible, and highly appraised method in patients undergoing NAST. Magseed® marking may be used with subsequent CESM to evaluate tumor response to NAST, reducing the workload of MRI services and facilitating scheduling.

## Supplementary Information

Below is the link to the electronic supplementary material.

Supplementary file1 (DOCX 94 KB)

## Data Availability

The datasets generated and/or analyzed during the current study are available from the corresponding author upon reasonable request.

## References

[1] Nederend J, Duijm LEM, Louwman MWJ et al (2012) Impact of transition from analog screening mammography to digital screening mammography on screening outcome in The Netherlands: a population-based study. Ann Oncol 23:3098–3103. 10.1093/ANNONC/MDS146 22745215 10.1093/annonc/mds146

[2] Tabár L, Dean PB (2010) A new era in the diagnosis and treatment of breast cancer. Breast J. 10.1111/J.1524-4741.2010.00992.X 21050302 10.1111/j.1524-4741.2010.00992.x

[3] Wazir U, Mokbel K (2022) De-escalation of breast cancer surgery following neoadjuvant systemic therapy. Eur J Breast Health 18:6–12. 10.4274/ejbh.galenos.2021.2021-5-4 35059585 10.4274/ejbh.galenos.2021.2021-5-4 PMC8734515

[4] Hadar T, Koretz M, Nawass M, Allweis TM (2021) Innovative standards in surgery of the breast after neoadjuvant systemic therapy. Breast Care (Basel) 16:590–597. 10.1159/000520051 35087362 10.1159/000520051 PMC8739938

[5] Chan BKY, Wiseberg-Firtell JA, Jois RHS et al (2015) Localization techniques for guided surgical excision of non-palpable breast lesions. Cochrane Database Syst Rev. 10.1002/14651858.CD009206.PUB2 26718728 10.1002/14651858.CD009206.pub2 PMC8881987

[6] Banys-Paluchowski M, Kühn T, Masannat Y et al (2023) Localization techniques for non-palpable breast lesions: current status, knowledge gaps, and rationale for the MELODY study (EUBREAST-4/iBRA-NET, NCT 05559411). Cancers 15:1173. 10.3390/CANCERS15041173 36831516 10.3390/cancers15041173 PMC9954476

[7] Kapoor MM, Patel MM, Scoggins ME (2019) The wire and beyond: recent advances in breast imaging preoperative needle localization. RadioGraphics 39:1886–1906. 10.1148/RG.2019190041 31560614 10.1148/rg.2019190041

[8] Cheang E, Ha R, Thornton CM, Mango VL (2018) Innovations in image-guided preoperative breast lesion localization. Br J Radiol. 10.1259/BJR.20170740 29271240 10.1259/bjr.20170740 PMC6190760

[9] Somasundaram SK, Potter S, Elgammal S et al (2021) Impalpable breast lesion localisation, a logistical challenge: results of the UK iBRA-NET national practice questionnaire. Breast Cancer Res Treat 185:13–20. 10.1007/S10549-020-05918-6 32914355 10.1007/s10549-020-05918-6

[10] Sajid MS, Parampalli U, Haider Z, Bonomi R (2012) Comparison of radioguided occult lesion localization (ROLL) and wire localization for non-palpable breast cancers: a meta-analysis. J Surg Oncol 105:852–858. 10.1002/JSO.23016 22213057 10.1002/jso.23016

[11] Franceschini G, Mason EJ, Grippo C et al (2021) Image-guided localization techniques for surgical excision of non-palpable breast lesions: an overview of current literature and our experience with preoperative skin tattoo. J Pers Med 11:1–14. 10.3390/JPM11020099 10.3390/jpm11020099 PMC7913802 33557072

[12] Gera R, Tayeh S, Al-Reefy S, Mokbel K (2020) Evolving role of magseed in wireless localization of breast lesions: systematic review and pooled analysis of 1559 procedures. Anticancer Res 40:1809–1815. 10.21873/anticanres.14135 32234869 10.21873/anticanres.14135

[13] Martínez M, Jiménez S, Guzmán F et al (2022) Evaluation of axillary lymph node marking with Magseed® before and after neoadjuvant systemic therapy in breast cancer patients: MAGNET study. Breast J. 10.1155/2022/6111907 35855102 10.1155/2022/6111907 PMC9288346

[14] Mariscal Martínez A, Vives Roselló I, Salazar Gómez A et al (2021) Advantages of preoperative localization and surgical resection of metastatic axillary lymph nodes using magnetic seeds after neoadjuvant chemotherapy in breast cancer. Surg Oncol 36:28–33. 10.1016/J.SURONC.2020.11.013 33285433 10.1016/j.suronc.2020.11.013

[15] McCamley C, Ruyssers N, To H et al (2021) Multicentre evaluation of magnetic technology for localisation of non-palpable breast lesions and targeted axillary nodes. ANZ J Surg 91:2411–2417. 10.1111/ANS.17108 34405514 10.1111/ans.17108

[16] Hayes MK (2017) Update on preoperative breast localization. Radiol Clin North Am 55:591–603. 10.1016/J.RCL.2016.12.012 28411682 10.1016/j.rcl.2016.12.012

[17] Al-Mousa DS (2023) Contrast enhanced mammography: another step forward in reducing breast cancer mortality. Acad Radiol 30:2252–2253. 10.1016/J.ACRA.2023.08.004 37633817 10.1016/j.acra.2023.08.004

[18] Iotti V, Ravaioli S, Vacondio R et al (2017) Contrast-enhanced spectral mammography in neoadjuvant chemotherapy monitoring: a comparison with breast magnetic resonance imaging. Breast Cancer Res. 10.1186/S13058-017-0899-1 28893303 10.1186/s13058-017-0899-1 PMC5594558

[19] Barra FR, Sobrinho AB, Barra RR et al (2018) Contrast-enhanced mammography (CEM) for detecting residual disease after neoadjuvant chemotherapy: a comparison with breast magnetic resonance imaging (MRI). Biomed Res Int. 10.1155/2018/8531916 30533440 10.1155/2018/8531916 PMC6250019

[20] Xiang W, Rao H, Zhou L (2020) A meta-analysis of contrast-enhanced spectral mammography versus MRI in the diagnosis of breast cancer. Thorac Cancer 11:1423–1432. 10.1111/1759-7714.13400 32233072 10.1111/1759-7714.13400 PMC7262891

[21] Tang S, Xiang C, Yang Q (2020) The diagnostic performance of CESM and CE-MRI in evaluating the pathological response to neoadjuvant therapy in breast cancer: a systematic review and meta-analysis. Br J Radiol. 10.1259/BJR.20200301 32574075 10.1259/bjr.20200301 PMC7446000

[22] Kaiyin M, Lingling T, Leilei T et al (2023) Head-to-head comparison of contrast-enhanced mammography and contrast-enhanced MRI for assessing pathological complete response to neoadjuvant therapy in patients with breast cancer: a meta-analysis. Breast Cancer Res Treat 202:1–9. 10.1007/S10549-023-07034-7 37615793 10.1007/s10549-023-07034-7

[23] Patel BK, Hilal T, Covington M et al (2018) Contrast-enhanced spectral mammography is comparable to MRI in the assessment of residual breast cancer following neoadjuvant systemic therapy. Ann Surg Oncol 25:1350–1356. 10.1245/S10434-018-6413-X/METRICS 29516362 10.1245/s10434-018-6413-x

[24] van Nijnatten TJA, Morscheid S, Baltzer PAT et al (2024) Contrast-enhanced breast imaging: current status and future challenges. Eur J Radiol 171:111312. 10.1016/j.ejrad.2024.111312 38237520 10.1016/j.ejrad.2024.111312

[25] Malherbe F, Roodt L, Noor F et al (2022) Magseed placement before neoadjuvant chemotherapy to facilitate subsequent breast-conserving surgery—a single-centre audit. South Afr J Surg 60:109–114. 10.17159/2078-5151/SAJS3679 35851364

[26] Mariscal Martínez A, Iglesias Bravo E, Peris Alvà H et al (2024) Contrast-enhanced mammography and magnetic seed localization for the detection of residual disease in breast cancer after neoadjuvant therapy. Radiologia. 10.1016/j.rx.2024.04.003 10.1016/j.rxeng.2024.04.001 39426811

[27] Eisenhauer EA, Therasse P, Bogaerts J et al (2009) New response evaluation criteria in solid tumours: revised RECIST guideline. Eur J Cancer 45:228–247. 10.1016/j.ejca.2008.10.026 19097774 10.1016/j.ejca.2008.10.026

[28] Miller ME, Patil N, Li P et al (2021) Hospital system adoption of magnetic seeds for wireless breast and lymph node localization. Ann Surg Oncol 28:3223–3229. 10.1245/S10434-020-09311-X 33170457 10.1245/s10434-020-09311-x

[29] Crèvecoeur J, Jossa V, Di Bella J et al (2023) Clinical experience of the Magseed® magnetic marker to localize non-palpable breast lesions: a cohort study of 100 consecutive cases. Gland Surg 12:566–576. 10.21037/GS-22-552/COIF 37284712 10.21037/gs-22-552 PMC10240439

[30] Powell M, Gate T, Kalake O et al (2021) Magnetic seed localization (Magseed) for excision of impalpable breast lesions—the North Wales experience. Breast J 27:529–536. 10.1111/tbj.14232 33855763 10.1111/tbj.14232

[31] Price ER, Khoury AL, Esserman LJ et al (2018) Initial clinical experience with an inducible magnetic seed system for preoperative breast lesion localization. AJR Am J Roentgenol 210:913–917. 10.2214/AJR.17.18345 29446680 10.2214/AJR.17.18345

[32] Zacharioudakis K, Down S, Bholah Z et al (2019) Is the future magnetic? Magseed localisation for non palpable breast cancer. A multi-centre non randomised control study. Eur J Surg Oncol 45:2016–2021. 10.1016/J.EJSO.2019.06.035 31288944 10.1016/j.ejso.2019.06.035

[33] Liang DH, Black D, Yi M et al (2022) Clinical outcomes using magnetic seeds as a non-wire, non-radioactive alternative for localization of non-palpable breast lesions. Ann Surg Oncol 29:3822–3828. 10.1245/S10434-022-11443-1 35233742 10.1245/s10434-022-11443-1 PMC11910204

[34] D’Angelo A, Trombadori CML, Caprini F et al (2022) Efficacy and accuracy of using magnetic seed for preoperative non-palpable breast lesions localization: our experience with Magseed. Curr Oncol 29:8468–8474. 10.3390/CURRONCOL29110667 36354727 10.3390/curroncol29110667 PMC9689792

[35] Jakub JW, Gray RJ, Degnim AC et al (2010) Current status of radioactive seed for localization of non palpable breast lesions. Am J Surg 199:522–528. 10.1016/J.AMJSURG.2009.05.019 19954769 10.1016/j.amjsurg.2009.05.019

[36] Kaufmann M, Von Minckwitz G, Mamounas EP et al (2012) Recommendations from an international consensus conference on the current status and future of neoadjuvant systemic therapy in primary breast cancer. Ann Surg Oncol 19:1508–1516. 10.1245/S10434-011-2108-2 22193884 10.1245/s10434-011-2108-2

[37] Jalloul M, Miranda-Schaeubinger M, Noor AM et al (2023) MRI scarcity in low- and middle-income countries. NMR Biomed 36:e5022. 10.1002/NBM.5022 37574441 10.1002/nbm.5022

[38] OECD, European Union (2018) Availability and use of diagnostic technologies, in health at a glance: Europe 2018: state of Health in the EU Cycle. OECD Paris/European Union, Brussels

[39] Mann RM, Cho N, Moy L (2019) Breast MRI: state of the art. Radiology 292:520–536. 10.1148/RADIOL.2019182947 31361209 10.1148/radiol.2019182947

[40] Phillips J, Steinkeler J, Talati K et al (2018) Workflow considerations for incorporation of contrast-enhanced spectral mammography into a breast imaging practice. J Am Coll Radiol 15:881–885. 10.1016/J.JACR.2018.02.012 29606631 10.1016/j.jacr.2018.02.012

[41] Jochelson MS, Lobbes MBI (2021) Contrast-enhanced Mammography: state of the art. Radiology 299:36–48. 10.1148/RADIOL.2021201948 33650905 10.1148/radiol.2021201948 PMC7997616

[42] Tayeh S, Gera R, Perry N et al (2020) The use of magnetic seeds and radiofrequency identifier tags in breast surgery for non-palpable lesions. Anticancer Res 40:315–321. 10.21873/ANTICANRES.13955 31892582 10.21873/anticanres.13955

[43] Schermers B, van der Hage JA, Loo CE et al (2017) Feasibility of magnetic marker localisation for non-palpable breast cancer. Breast 33:50–56. 10.1016/J.BREAST.2017.03.003 28282587 10.1016/j.breast.2017.03.003

[44] Micha AE, Sinnett V, Downey K et al (2021) Patient and clinician satisfaction and clinical outcomes of Magseed compared with wire-guided localisation for impalpable breast lesions. Breast Cancer 28:196–205. 10.1007/s12282-020-01149-1 32974810 10.1007/s12282-020-01149-1 PMC7796883

