# Supplementary material for: Reliability of Magseed® marking before neoadjuvant systemic therapy with subsequent contrast-enhanced mammography in patients with non-palpable breast cancer lesions after treatment: the MAGMA study
Source: Breast Cancer Res Treat. 2024 Jun 20;208(1):133–43. doi: 10.1007/s10549-024-07407-6 (PMC11452456; doi:10.1007/s10549-024-07407-6)
Supplement: Supplementary file 1 — Supplementary file1 (DOCX 94 KB) [file 10549_2024_7407_MOESM1_ESM.docx]

# Supplementary Materials for the Submission of “Reliability of Magseed® Marking Before Neoadjuvant Systemic Therapy With Subsequent Contrast-Enhanced Mammography in Patients With Non-Palpable Breast Cancer Lesions After Treatment: The MAGMA Study” by Iglesias Bravo et al.

# Supplementary Tables

| **Table S1.** Magseed®-related variables and affected lymph nodes at baseline. | |
| --- | --- |
| Positive lymph nodes, n (%) n=109 |  |
| Yes | 59 (54.13) |
| No | 50 (45.87) |
| Number of positive lymph nodes, n (%) n=59 |  |
| 1 | 35 (59.32) |
| 2 | 7 (11.86) |
| 3 | 8 (13.56) |
| 4 | 8 (13.56) |
| 5 | 1 (1.69) |
| Marked positive lymph nodes, n (%) n=59 |  |
| Yes | 46 (77.97) |
| No | 13 (22.03) |
| Lymph node marker, n (%) n=46 |  |
| Magseed® | 42 (91.31) |
| Other seeds | 4 (8.69) |

| **Table S2.** Magseed® retrieval from axillary lymph node tissues, n=109. | |
| --- | --- |
| **Axillary lymph nodes** |  |
| Magseed® retrieval, n (%) n=42 | 42 (100) |
| Correspondence between marked LN and sentinel LN, n=42 |  |
| Yes | 28 (66.66) |
| No | 14 (33.37) |
| LN, lymph node; CESM, contrast-enhanced spectral mammography; CI, confidence interval. | |

| **Table S3.** Histopathological characteristics of dissected breast tumors, n (%) n=109 | |
| --- | --- |
|  | **Dissected tumors n=106**^a^ |
| Histological type |  |
| Ductal | 98 (92.45) |
| Lobular | 6 (5.66) |
| Others | 2 (1.89) |
| Tumoral grade |  |
| I | 8 (7.55) |
| II | 61 (57.55) |
| III | 37 (34.91) |
| Tumoral phenotype |  |
| HER2 | 10 (9.43) |
| Luminal A | 7 (6.60) |
| Luminal B | 45 (42.45) |
| Luminal B HER2 | 19 (17.92) |
| Triple negative | 25 (23.58) |
| T, n=104^b^ |  |
| Tis | 7 (6.73) |
| T0 | 22 (21.15) |
| T1 | 46 (44.23) |
| T2 | 25 (24.04) |
| T3 | 4 (3.85) |
| T4 | 0 (0.00) |
| Any T | 0 (0.00) |
| N, n=104^b^ |  |
| N0 | 64 (61.54) |
| N1 | 33 (31.73) |
| N2 | 3 (2.88) |
| N3 | 4 (3.85) |
| Any N | 0 (0.00) |
|  |  |
| Miller-Payne Grade |  |
| G1 | 4 (3.77) |
| G2 | 8 (7.55) |
| G3 | 37 (34.91) |
| G4 | 23 (21.70) |
| G5 | 34 (32.08) |
| ^a^Missing tissues due to specimen deterioration causing Magseed® loss in the operating room before radiological examination.  ^b^Missing data in two samples. | |

| **Table S4.** Patients' satisfaction survey, n (%) n=65^a^. | |
| --- | --- |
| **1.** The use of the tumor localization technique with the Magseed® magnetic seed placed before starting neoadjuvant chemotherapy treatment avoids the need to place a surgical harpoon on the same day of surgery. How effective do you find this localization technique? (Please choose a score between 1 and 7). | |
| 1 | 0 (0) |
| 2 | 0 (0) |
| 3 | 0 (0) |
| 4 | 0 (0) |
| 5 | 2 (3.1) |
| 6 | 8 (12.3) |
| 7 | 55 (84.6) |
| **2.** If you have experienced discomfort/pain during or after the Magseed®/harpoon placement, please rate the intensity on a 1-7 scale (1, no pain; 7, severe pain). | |
| 1 | 40 (61.5) |
| 2 | 12 (18.5) |
| 3 | 6 (9.2) |
| 4 | 3 (4.6) |
| 5 | 1 (1.5) |
| 6 | 1 (1.5) |
| 7 | 2 (3.1) |
| 3. If the procedure has caused any adverse effects, please rate the intensity level (1, mild intensity; 7, high intensity). | |
| 1 | 46 (70.8) |
| 2 | 14 (21.5) |
| 3 | 3 (4.6) |
| 4 | 0 (0) |
| 5 | 1 (1.5) |
| 6 | 0 (0) |
| 7 | 1 (1.5) |
| 4. Overall, your satisfaction with the entire procedure is: (please choose a score between 1 and 7). | |
| 1 | 0 (0) |
| 2 | 0 (0) |
| 3 | 0 (0) |
| 4 | 1 (1.5) |
| 5 | 0 (0) |
| 6 | 6 (9.2) |
| 7 | 58 (89.2) |
| ^a^Data were missing for 43 (39.8%) patients who did not answer the survey. | |

# Supplementary Figures


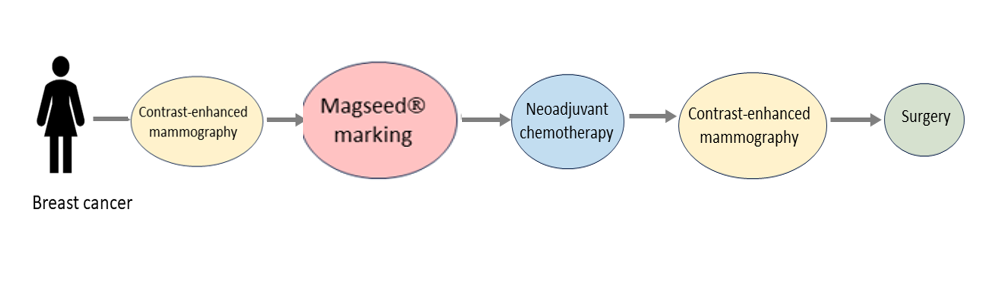


**Figure S1.** Diagram of the study design. NAST, neoadjuvant chemotherapy.

Recruited patients

N=131

Evaluable patients

N=109

Excluded, n=22

Not meeting inclusion criteria, n=20

Not evaluable due to loss to follow up, n=2

**Figure S2.** Flow chart of study patients.
